# Supplementary material for: Selective N-terminal acylation of peptides and proteins with a Gly-His tag sequence
Source: Nat Commun. 2018 Aug 17;9:3307. doi: 10.1038/s41467-018-05695-3 (PMC6098153; doi:10.1038/s41467-018-05695-3)
Supplement: Supplementary file 1 — Supplementary Information [file 41467_2018_5695_MOESM1_ESM.pdf]

Supplementary Information

Selective N-terminal acylation of peptides and proteins with a Gly-His sequence

Martos-Maldonado et al.

Table of contents

Supplementary Figure 1 HSQC and HMBC NMR of gluconoylated nonapeptide **9**
Supplementary Figure 2 Reaction of peptide **15** with GDL
Supplementary Figure 3 Other acylating agents evaluated
Supplementary Figure 4 Stability of 4-methoxyphenyl 2-azidoacetate **18**
Supplementary Figure 5 Progress in acylation of peptide **15** with **18**
Supplementary Figure 6 ESI MS/MS data of azidoacetyl-GHHHHHH-Beltide-1 (**15Az**)
Supplementary Figure 7 Analysis of acylation sites for reaction of **15** and **18** by trypsin digestion
Supplementary Figure 8 Peak identification for trypsin data in Supplementary Fig. 7
Supplementary Figure 9 Progress in degluconoylation of peptide **15Gdl**
Supplementary Figure 10 Degluconoylation of GH_6-EGFP
Supplementary Figure 11 Stability of acylated peptide **15Az**
Supplementary Figure 12 Stability of acylated GH_6-EGFP
Supplementary Figure 13 Optimization of the acylation of GH_6-EGFP with **18**
Supplementary Figure 14 MS spectra of biotinylated GH_6-EGFP after digestion with trypsin
Supplementary Figure 15 Control experiments of $GSSH_6$-EGFP and GSH-EGFP treated with **18**
Supplementary Figure 16 Control experiments using GSH-EGFP and Ac-GH_6-NH_2 or imidazole
Supplementary Figure 17 Deconvoluted ESI-TOF spectrum of the reaction of GH_6-BIR2 with **18**
Supplementary Figure 18 One-step biotinylation of GH_6-EGFP with **21**
Supplementary Figure 19 Processing of biotinylated GH_6-SUMO with SUMO protease
Supplementary Figure 20 NMR spectroscopic analysis of potential N-acyl imidazole formation
Supplementary Figure 21 Spectrophotometric analysis of potential N-acyl imidazole formation
Supplementary Figure 22 Hydrolysis of **18** in the presence of different imidazoles
Supplementary Figure 23 ^{1}H- and ^{13}C-NMR spectra of ester **16**
Supplementary Figure 24 ^{1}H- and ^{13}C-NMR spectra of ester **17**
Supplementary Figure 25 ^{1}H- and ^{13}C-NMR spectra of ester **18**
Supplementary Figure 26 ^{1}H- and ^{13}C-NMR spectra of the biotin reagent **20**
Supplementary Figure 27 COSY and HSQC NMR spectra of the biotin reagent **20**
Supplementary Figure 28 Uncropped versions of the gels and blots depicted in Figures 4 and 5

Supplementary Table 1 Optimization of the acylation of GH6-SUMO with ester **18**
Supplementary Table 2 Purity and masses of all peptides synthesized
Supplementary Table 3 Mass spectrometry data of acylated Beltide-1 derivatives

Supplementary Methods
Protein Sequences
Reaction of GH6-EGFP with biotin derivative **21**

Supplementary Figures

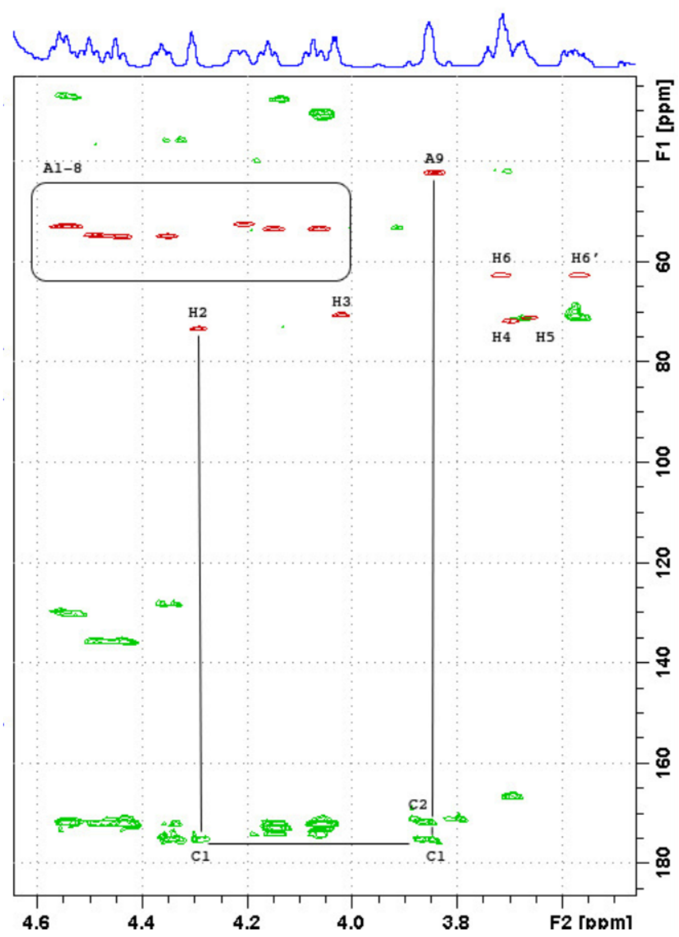

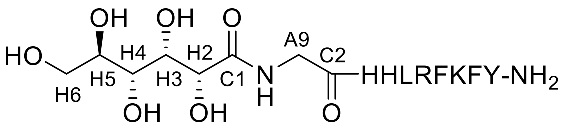

Supplementary Figure 1. HSQC and HMBC NMR of gluconoylated nonapeptide **9** (D-gluconoyl-GHHLRFKFY-NH_2). An overlay of 500 MHz 2D-spectra, HSQC (marked in red) and HMBC (marked in green), allows determination of connectivity over five bonds (indicated by black connecting line). This makes it possible to conclude that gluconoylation has taken place at the N-terminus of the peptide. Assignments A1-A9 represent the C^{α}-H^{α} single bond HSQC correlations of the peptide backbone. Correlation A9 is assigned to the N-terminal Gly based on the characteristic upfield shift compared to the other amino acids, as well as from the characteristic ^{1}H-NMR absorption. Assignments H2-H6 represent the carbohydrate C-H HSQC correlations of the gluconoyl group. It can be seen that the Gly H^{α} proton (A9) couples to two different carbons (C1 and C2) in the carbonyl area through multiple-bond HMBC correlations. Furthermore, one of the carbonyls (C1) couples to a proton in the carbohydrate area (C1 to H2). The overlay of these two spectra thus enables assignment of connectivity between proton H2 of the gluconoyl group to the carbonyl carbon C2 of the N-terminal Gly. This unequivocally shows that 1,5-gluconolactone has reacted with the N-terminal Gly in peptide **9**.

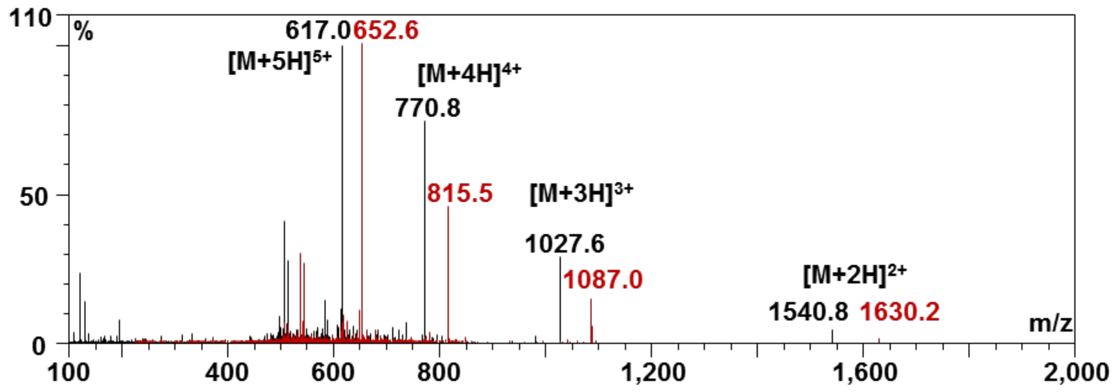

Supplementary Figure 2. Reaction of peptide **15** with GDL. Mass spectra of peptide **15** before (black) and after (red) being treated at a concentration of 1 mM with 100 equivalents of GDL in 200 mM HEPES buffer at pH 7.5 at room temperature for 1 h.

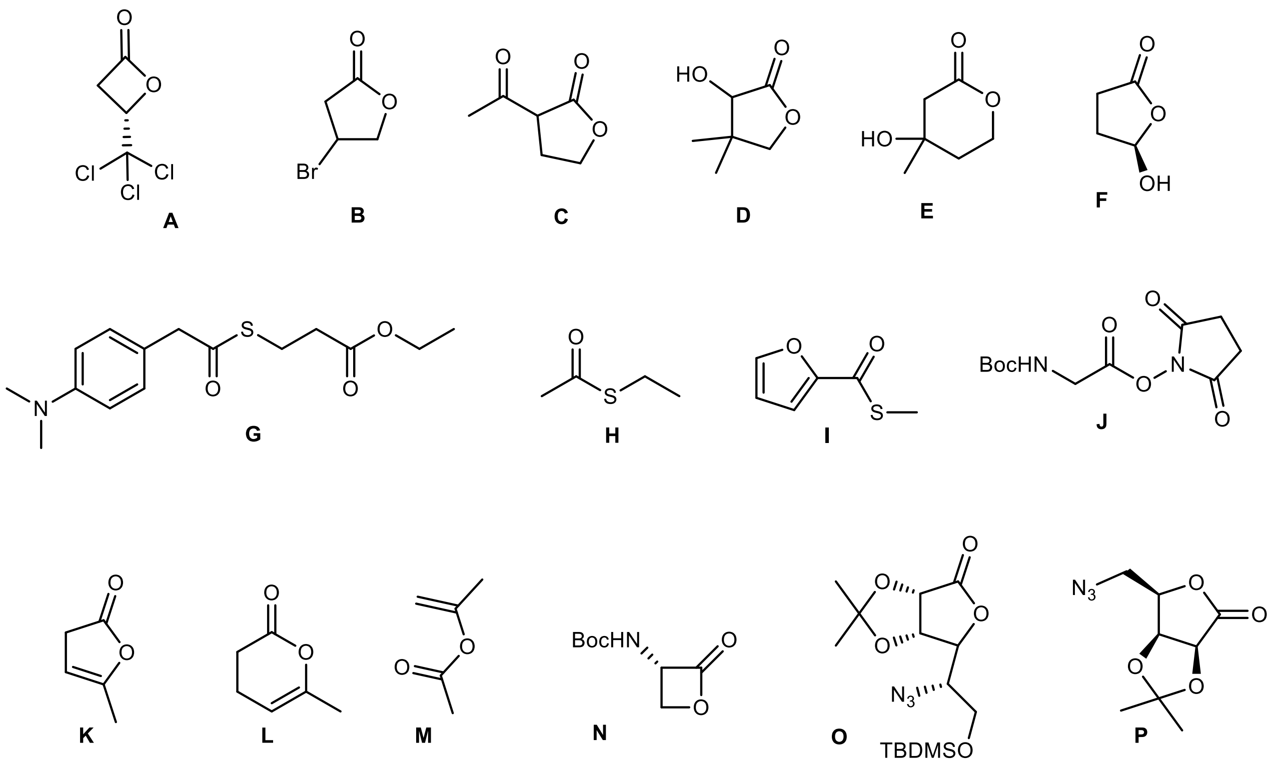

Supplementary Figure 3. Other acylating agents evaluated. A: (*S*)-4-trichloromethyl-butyric-β-lactone, B: 2-bromo-γ-butyrolactone, C: α-acetylbutyrolactone, D: pantolactone, E: mevalonolactone, F: 4-hydroxybutyrolactone, G: (3-dimethylamino-phenylacetyl)-ethylthiopropionate, H: Ethyl thioacetate, I: Methyl furan-2-thiocarboxylate, J: Boc-Gly-OSu, K: α-angelica-lactone, L: 3,4-Dihydro-6-methyl-2H-pyran-2-one, M: Isopropenyl acetate, N: Boc-L-Serine-β-lactone, O: 5-Azido-6-O-(tert-butyldimethylsilyl)-2,3-O-isopropylidene L-gulono-1,4-lactone, P: 5-azido-5-deoxy-2,3-isopropylidene-D lyxono- 1,4-lactone.

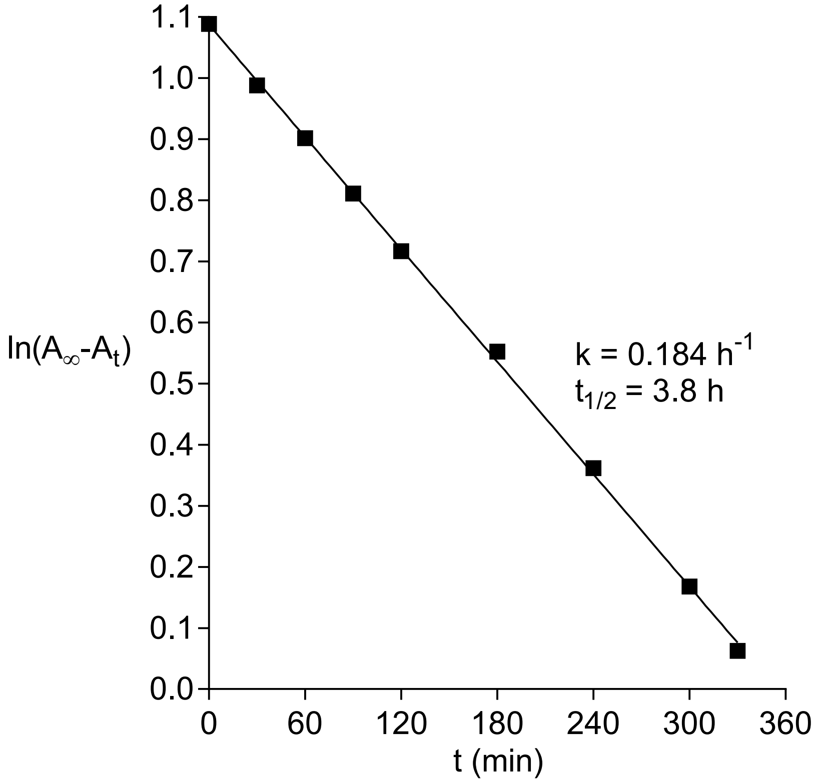

Supplementary Figure 4. Stability of 4-methoxyphenyl 2-azidoacetate **18**. Conditions: 4 °C, 200 mM HEPES buffer, pH 7.5. A 25 mM solution **18** in acetonitrile (0.05 mL) was added to 200 mM HEPES buffer, pH 7.5 (0.45 mL). UV spectra were acquired using a Jasco V-650 spectrophotometer. The absorption at 295 nm of 4-methoxyphenol was used in the kinetic determination. The curve is based on a single measurement.

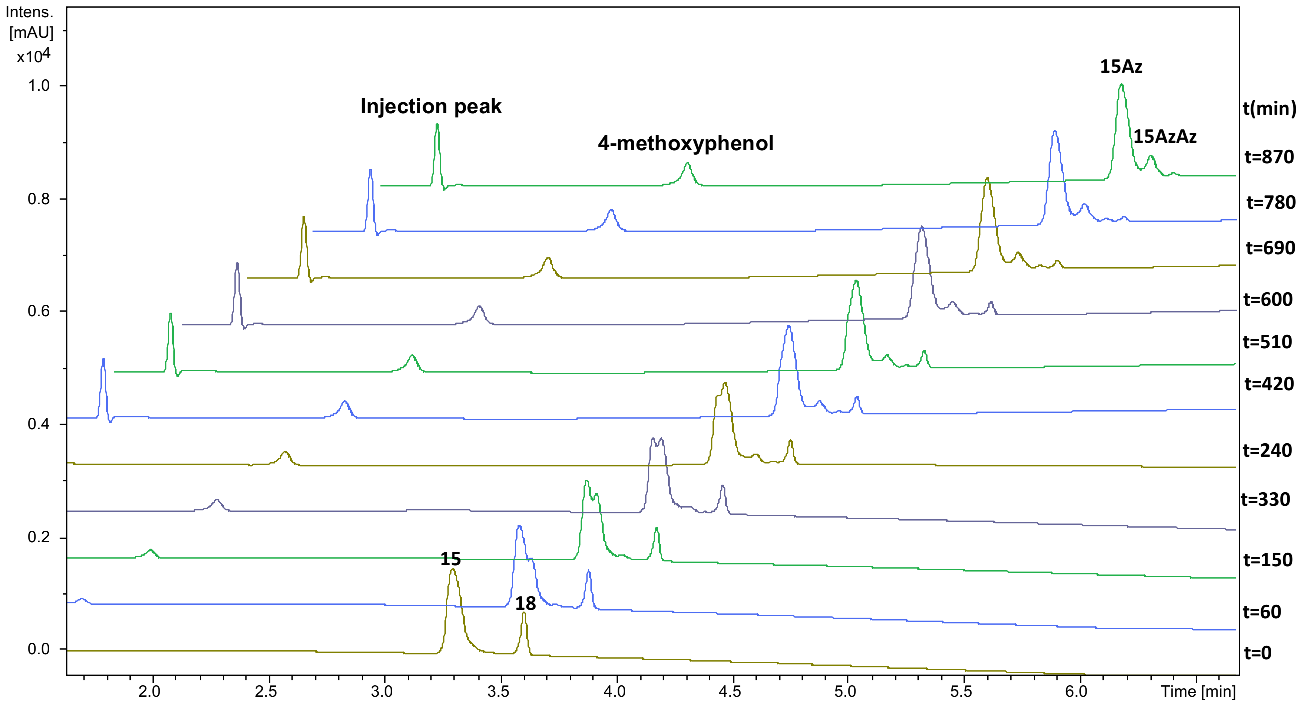

Supplementary Figure 5. Stacked overlay of HPLC measurements acquired over 24 h for reaction between GHHHHHH-Beltide-1 (**15**, retention time = 3.3 min) and 4-methoxyphenyl 2-azidoacetate (**18**, retention time = 3.6 min) in 200 mM HEPES buffer, pH 7.5, at 4 °C. The N-terminal mono-acylated product (**15Az**) and a minor di-acylated product (**15Az-Az**) have retention times of 3.35 min and 3.45 min, respectively. The reaction reached 50% conversion after approximately 4 h.

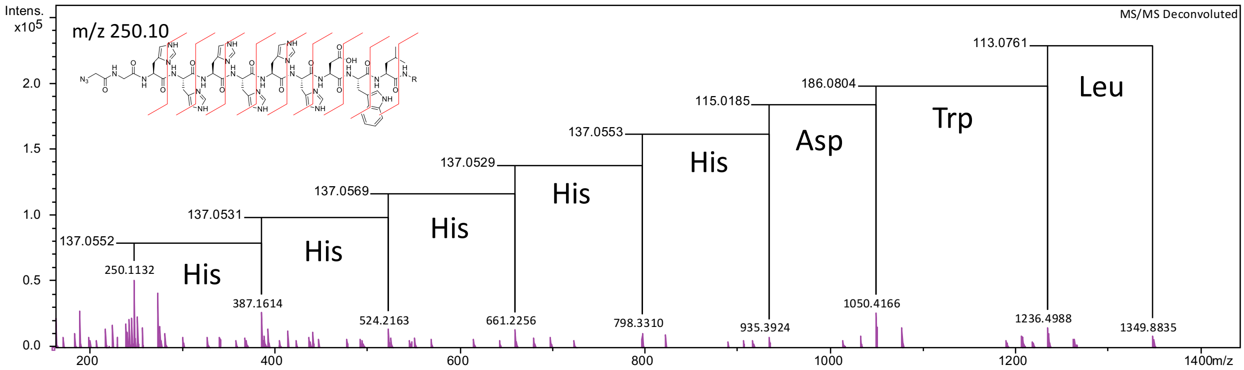

Supplementary Figure 6. N-terminal acylation of peptide **15** with **18**. ESI MS/MS data of azidoacetyl-Beltide-1 (**15Az**).

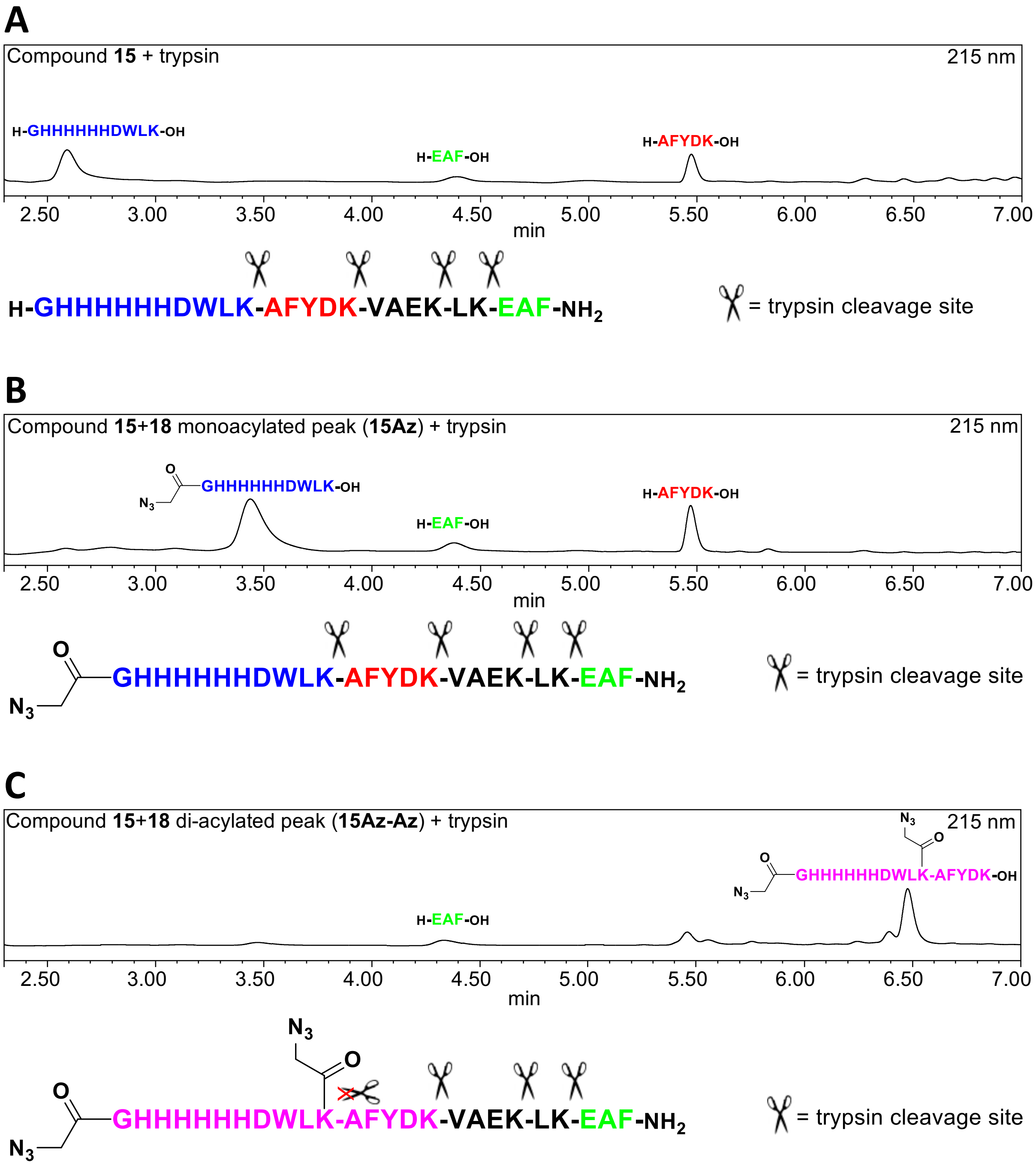

Supplementary Figure 7. Analysis of acylation sites for reaction of 15 and 18 by trypsin digestion. LC-MS after trypsin cleavage of A) **15** (control), B) main reaction product **15Az**, and C) minor di-acylated product **15Az-Az**. Indicated peak identities were confirmed by MS. The primary location of the second acylation site in **15Az-Az** was found to be at the lysine in closest proximity to the hexahistidine tag, as determined by the presence of a strong peak at 6.50 min, corresponding to a 16-mer peptide containing two 2-azidoacetyl groups.

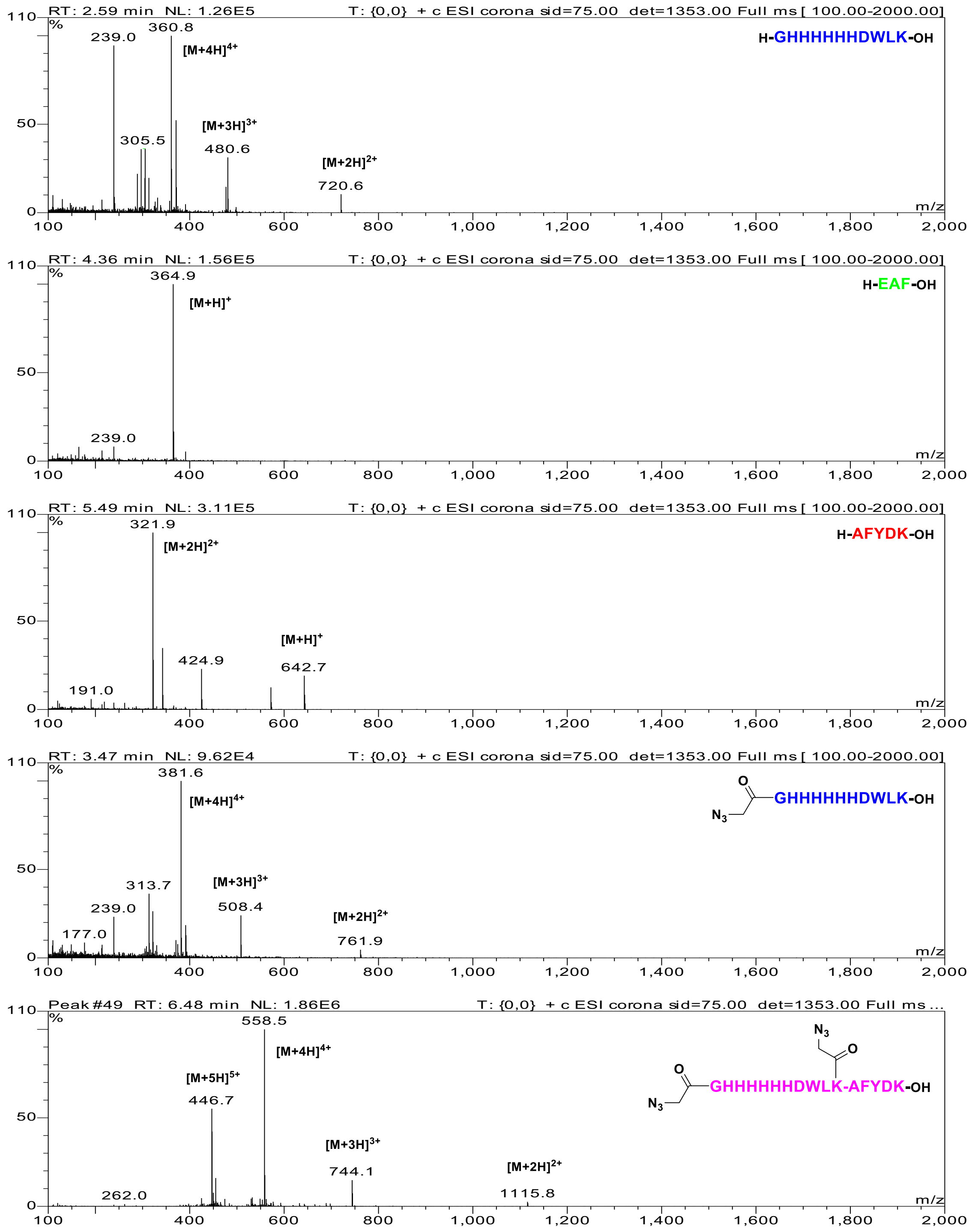

Supplementary Figure 8. Peak identification for trypsin data in Supplementary Fig. 7. Analytical LC-MS was performed on a Dionex Ultimate 3000 instrument with an C4 column (Higgins Analytical, Proto 300, 300 Å, 5 µm, 4.6x150 mm) coupled to a ESI-MS (MSQ Plus Mass Spectrometer, Thermo) using a linear gradient flow of water-acetonitrile containing 0.1% formic acid.

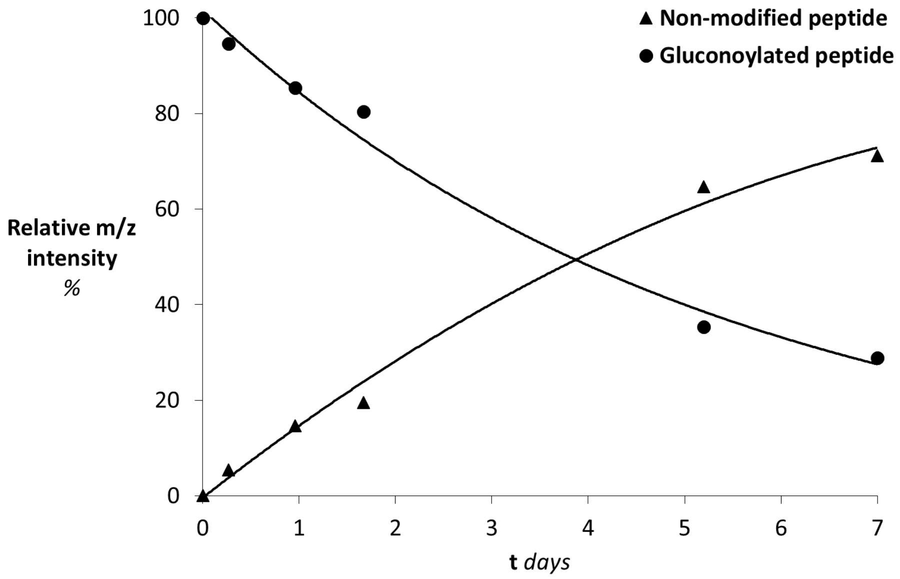

Supplementary Figure 9. Progress in degluconoylation of peptide **15Gdl**. Change in the relative m/z intensity ($[m/z_1/(m/z_1+m/z_2)]\cdot 100$) by ESI-MS of **15Gdl** and **15** with time at room temperature; the initial concentration was 500 µM gluconoylated GHHHHHH-Beltide-1 (**15Gdl**) in 50 mM phosphate buffer at pH 7.5. Curves are based on a single measurement.

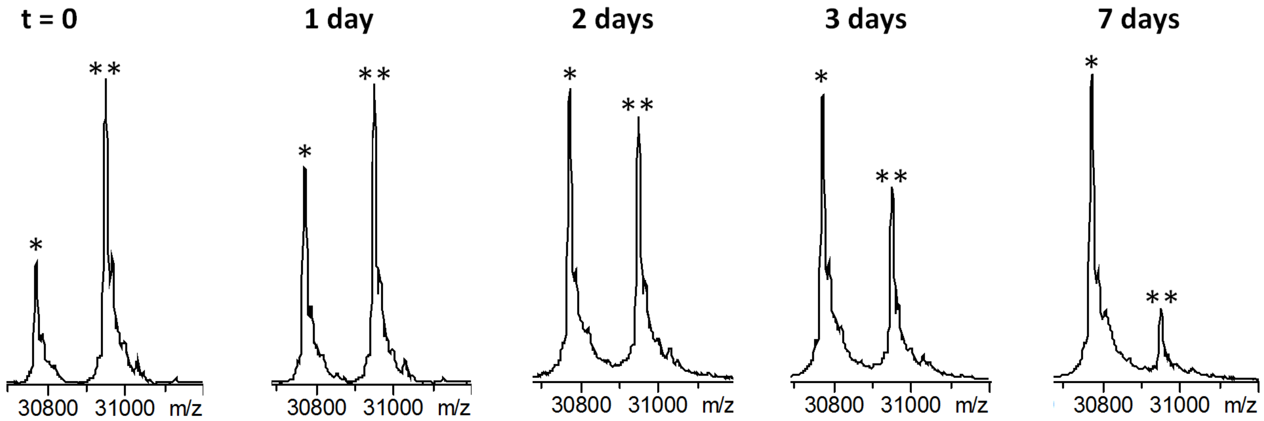

Supplementary Figure 10. Degluconoylation of GH_6-EGFP. Deconvoluted ESI-TOF spectra showing the reversibility of the gluconoylation of GH_6-EGFP. GDL was removed by spin-filtration and the protein (32.5 µM) was left in an aqueous solution of 50 mM phosphate buffer and 150 mM NaCl (pH 7.5) at room temperature. The protein was analyzed by LC-MS at given time points. The starting material (*) and gluconoylated product (**) are indicated.

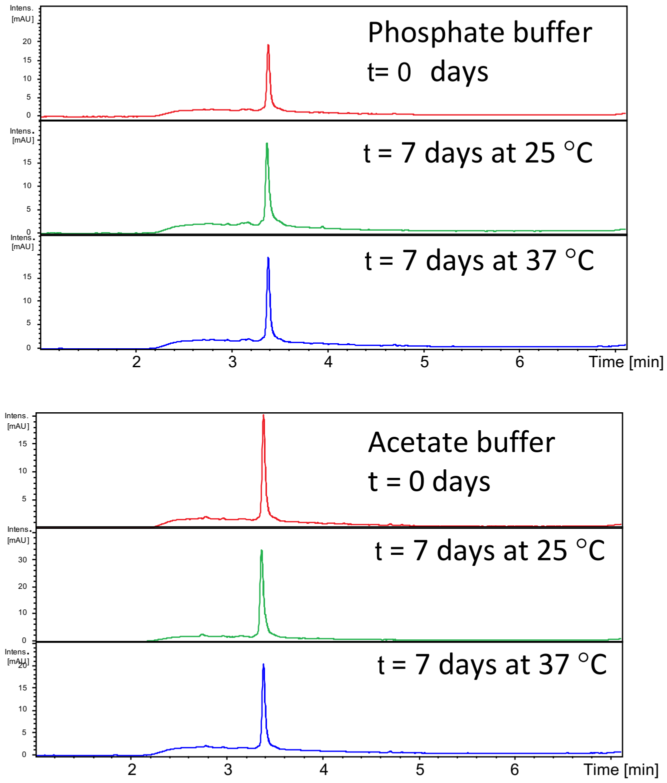

Supplementary Figure 11. Stability of acylated peptide **15Az**. Isolated peptide **15Az** was dissolved in phosphate buffered saline or in 10 mM acetate buffer, pH 4.7 , and then it was incubated for 7 days at 25 °C or at 37 °C. In no case was any deacylation visible by HPLC UV (215 nm) or MS detection.

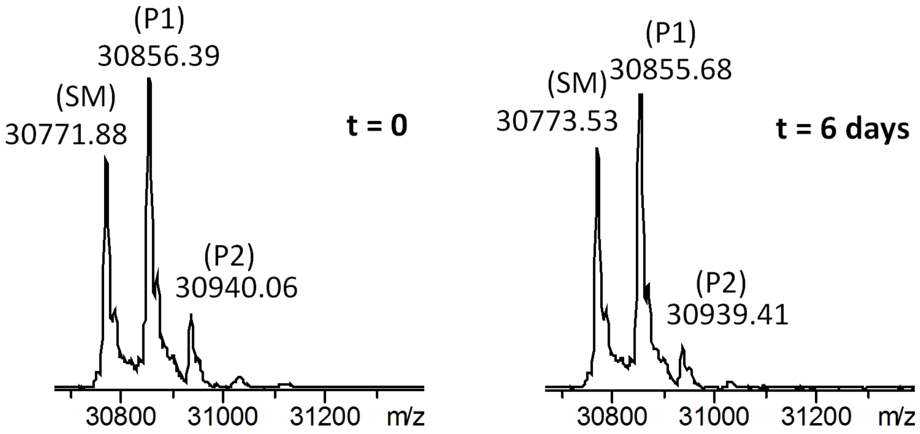

Supplementary Figure 12. Stability of acylated GH_6-EGFP. Deconvoluted ESI-TOF spectrum of GH_6-EGFP modified with 4-methoxyphenyl ester **18** after incubation at 4 °C for 6 days in an aqueous solution of 50 mM phosphate buffer and 150 mM NaCl (pH 7.5). For comparison, the spectrum of the protein obtained at day 0 is depicted as well. The unmodified protein is labeled SM, and species corresponding to the product mass are labeled P1 and P2; the number indicating the number of modifications introduced.

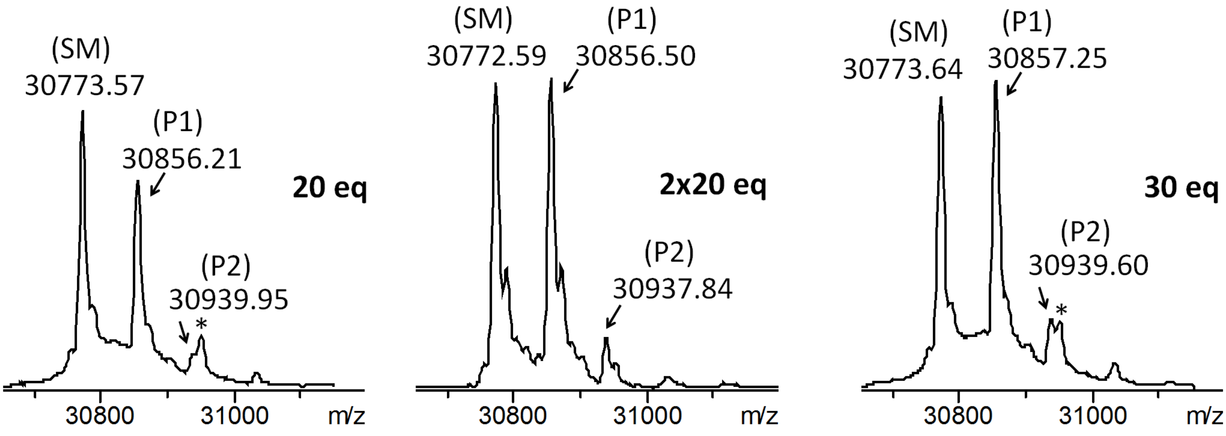

Supplementary Figure 13. Optimization of the acylation of GH_6-EGFP with **18**. Deconvoluted ESI-TOF spectra of the reaction of GH_6-EGFP at a concentration of 35 µM with 20–30 equiv. of 4-methoxyphenyl ester **18** in 200 mM HEPES buffer at pH 7.5 with ≤5 % acetonitrile at 4 °C for 1 day. As for the “20x20eq” reaction, a fresh portion of 20 equiv. of **18** was added the next day and the reaction was allowed to proceed at 4 °C for an additional day. The unmodified protein is labeled SM, and species corresponding to the product mass are labeled P1 and P2; the number indicating the number of modifications introduced. The asterix (*) indicates endogenously gluconoylated GH_6-EGFP, which was present in the starting material (calculated M_r 30952.03 Da).

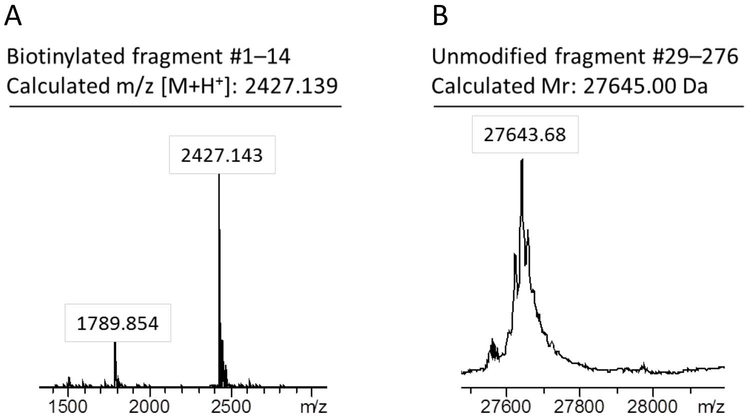

Supplementary Figure 14. MS spectra of biotinylated GH_6-EGFP after digestion with trypsin. *A:* MALDI-TOF spectrum of the N-terminal fragment consisting of amino acids #1–14. *B:* Deconvoluted ESI-TOF spectrum of fragment #29–276. The peak at m/z 1789.854 appears to be a decomposed or truncated version of the biotinylated fragment (which could be due to fragmentation during MS analysis, or due to an impurity precursor DBCO-PEG4-biotin **19**).

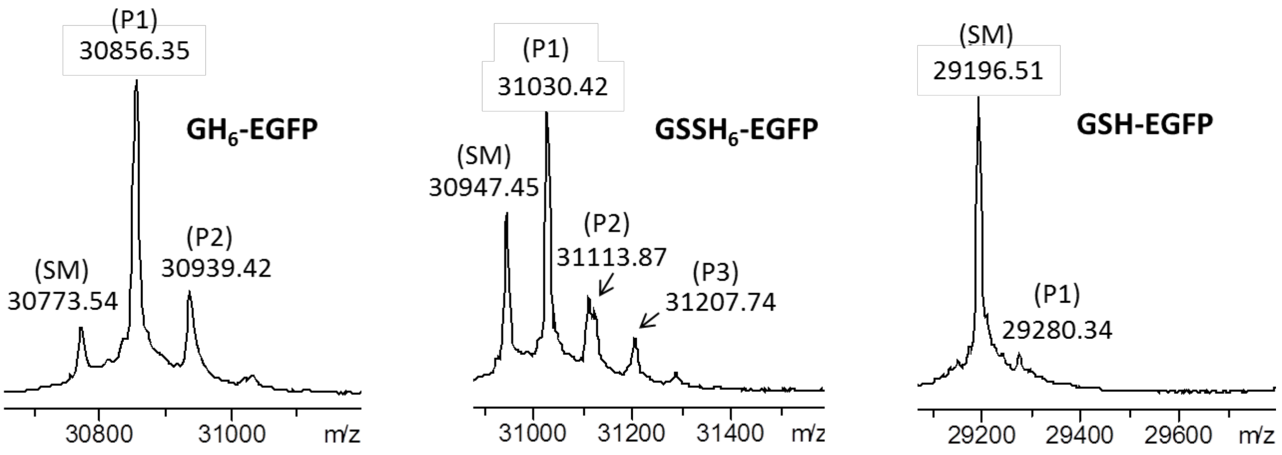

Supplementary Figure 15. Control experiments of GSSH$_6$-EGFP and GSH-EGFP treated with **18**. Deconvoluted ESI-TOF spectra of the control reaction of GSSH$_6$-EGFP and GSH-EGFP at a concentration of 35 µM with 40 equiv. of compound **18** in 200 mM HEPES buffer at pH 7.5 with 10 % acetonitrile at room temperature for 2 days. For comparison the ESI-TOF spectrum of GH$_6$-EGFP (also shown in Figure 4B) is depicted as well. Unmodified proteins are labeled SM, and species corresponding to the product mass are labeled P1, P2, and P3; the number indicating the number of modifications introduced.

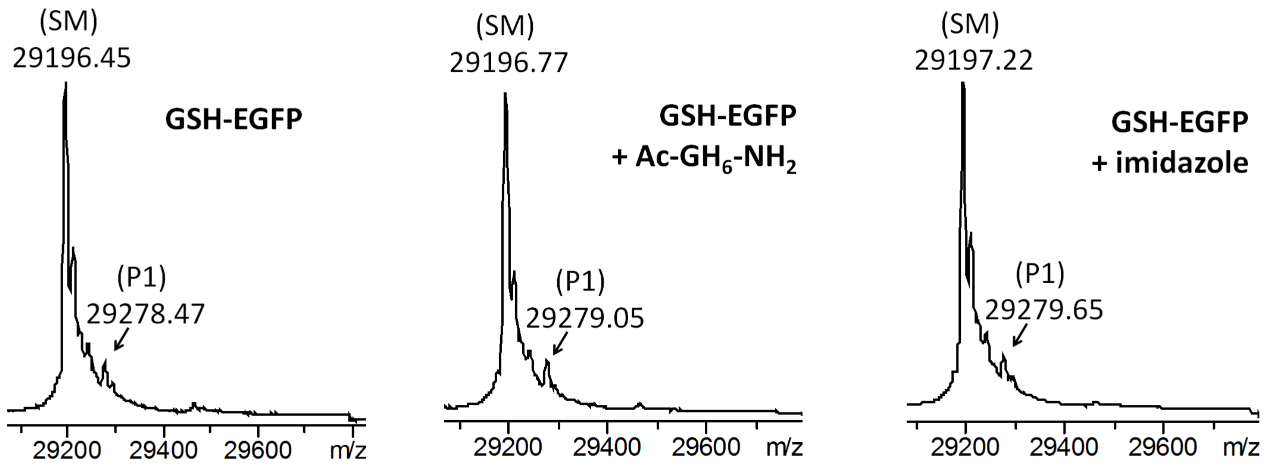

Supplementary Figure 16. Control experiments using GSH-EGFP and Ac-GH$_6$-NH$_2$ or imidazole. Deconvoluted ESI-TOF spectra of the control reaction of GSH-EGFP (32.5 µM) in the presence of a hexahistidine peptide (Ac-GH$_6$-NH$_2$) or imidazole with 40 equiv. of compound **18** in 200 mM HEPES buffer (pH 7.5) with 5 % acetonitrile at 4 °C for 1 day. An equimolar amount of Ac-GH$_6$-NH$_2$ and 6-fold excess of imidazole relative to protein were added, respectively. Unmodified protein is labeled SM, and the species corresponding to the product mass is labeled P1.

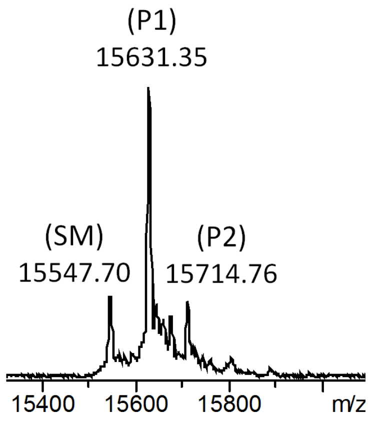

Supplementary Figure 17. Acylation of GH_6-BIR2 with **18**. Deconvoluted ESI-TOF spectrum of the reaction of GH_6-BIR2 at a concentration of 40 µM with 20 equiv. of 4-methoxyphenyl ester **18** in 50 mM NaH_2PO_4, 150 mM NaCl (pH 7.5) with 6 % acetonitrile at 4 °C for 1 day. Unmodified protein is labeled SM, and species corresponding to the product masses are labeled P1 and P2; the number indicating the number of modifications introduced.

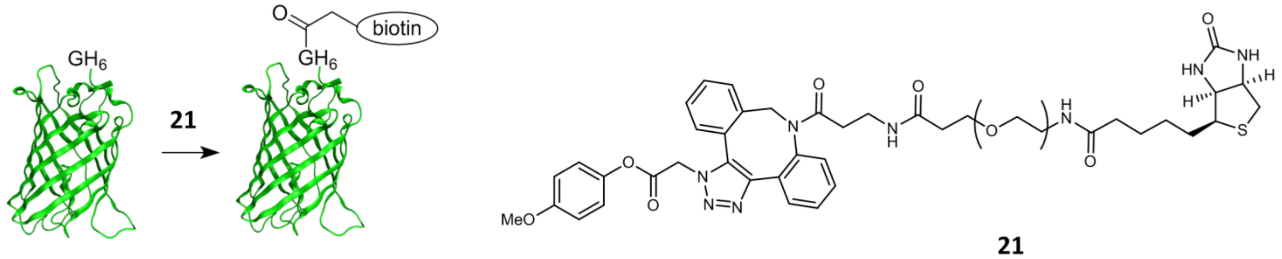

21

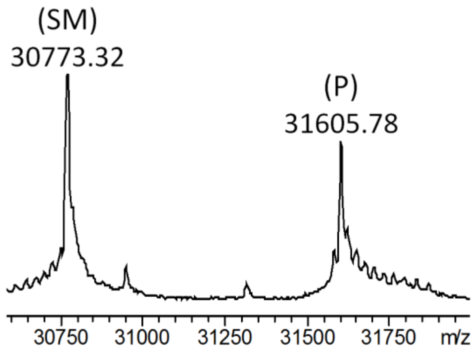

Supplementary Figure 18. One-step biotinylation of GH_6-EGFP with **21**. Deconvoluted ESI-TOF spectrum of the direct biotinylation of GH_6-EGFP via *in situ* formation of 4-methoxy phenyl ester **21**. Unmodified protein is labeled SM, and the species corresponding to the mono-labeled product mass is labeled P1.

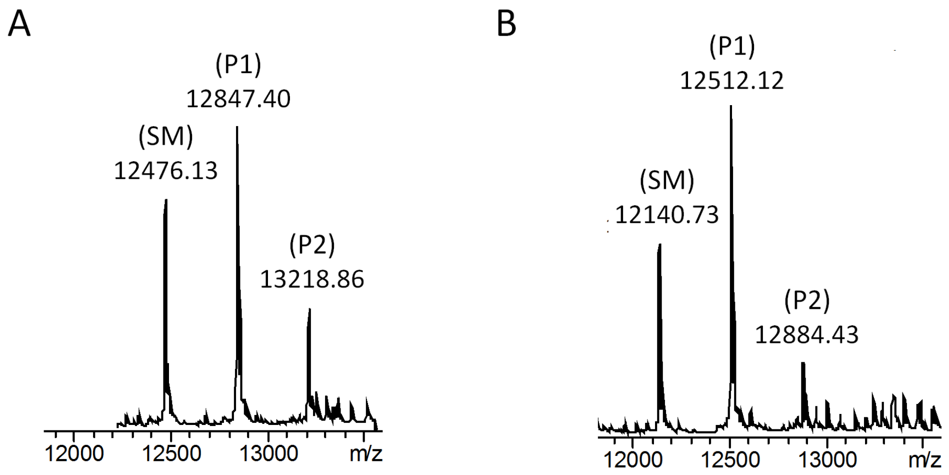

Supplementary Figure 19. Processing of biotinylated GH_6-SUMO with SUMO protease. A) Deconvoluted ESI-TOF MS spectrum of biotinylated GH_6-SUMO (from non-optimized reaction). The calculated increase in mass upon the addition of one biotin moiety is 371.15 Da. B) Deconvoluted ESI-TOF spectrum of the same sample treated with SUMO protease for 1 h at 30°C. Unmodified protein is labeled SM, and species corresponding to the product masses are labeled P1 and P2; the number indicating the number of modifications introduced. The mass shift after proteolytic cleavage of the last three amino acids (ATY) at the C-terminus is calculated to be 335.4 Da. All species, unmodified and biotinylated, were completely processed by the protease. Note that biotin reagent **20** was not removed before adding SUMO protease, which explains the slight increase in the relative amount of mono-biotinylated product in B) compared to A).

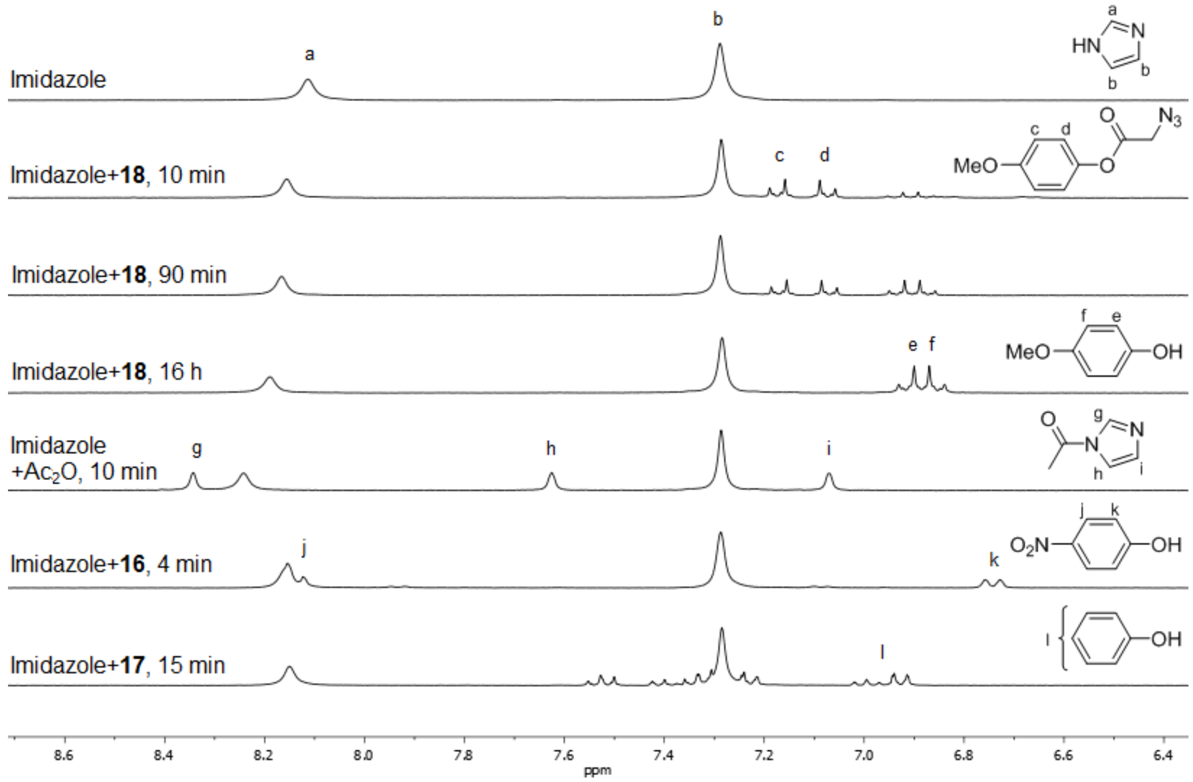

Supplementary Figure 20. NMR spectroscopic analysis of potential N-acyl imidazole formation at 25 °C. Three characteristic peaks originating from the N-acyl imidazole in the range 8.4-7.0 ppm were evident with acetic anhydride (g, h, and i in the lower panel), but they were not detected when **18** was used as the acylating agent. Peaks originating from imidazole (a, b), **18** (c, d), and 4-methoxyphenol (e, f) are indicated. A slight drift of imidazole H-2 (a) was due to liberation of acidic products of hydrolysis. A 100 mM phosphate buffer, pD 7.5, prepared by dissolution of trisodium phosphate in D_2O, followed by adjustment of pD with concentrated DCl in D_2O, was used. Imidazole (1.1 mg, final concentration of 25 mM) was dissolved in the buffer (0.63 mL). A CD_3CN (0.07 mL) solution containing **18** (100 mM, final concentration of 10 mM), acetic anhydride (100 mM, final concentration of 10 mM), or no acylating agent was added. Following rapid mixing, ^{1}H-NMR spectra were acquired using a Bruker Avance 300 MHz instrument at 300 K.

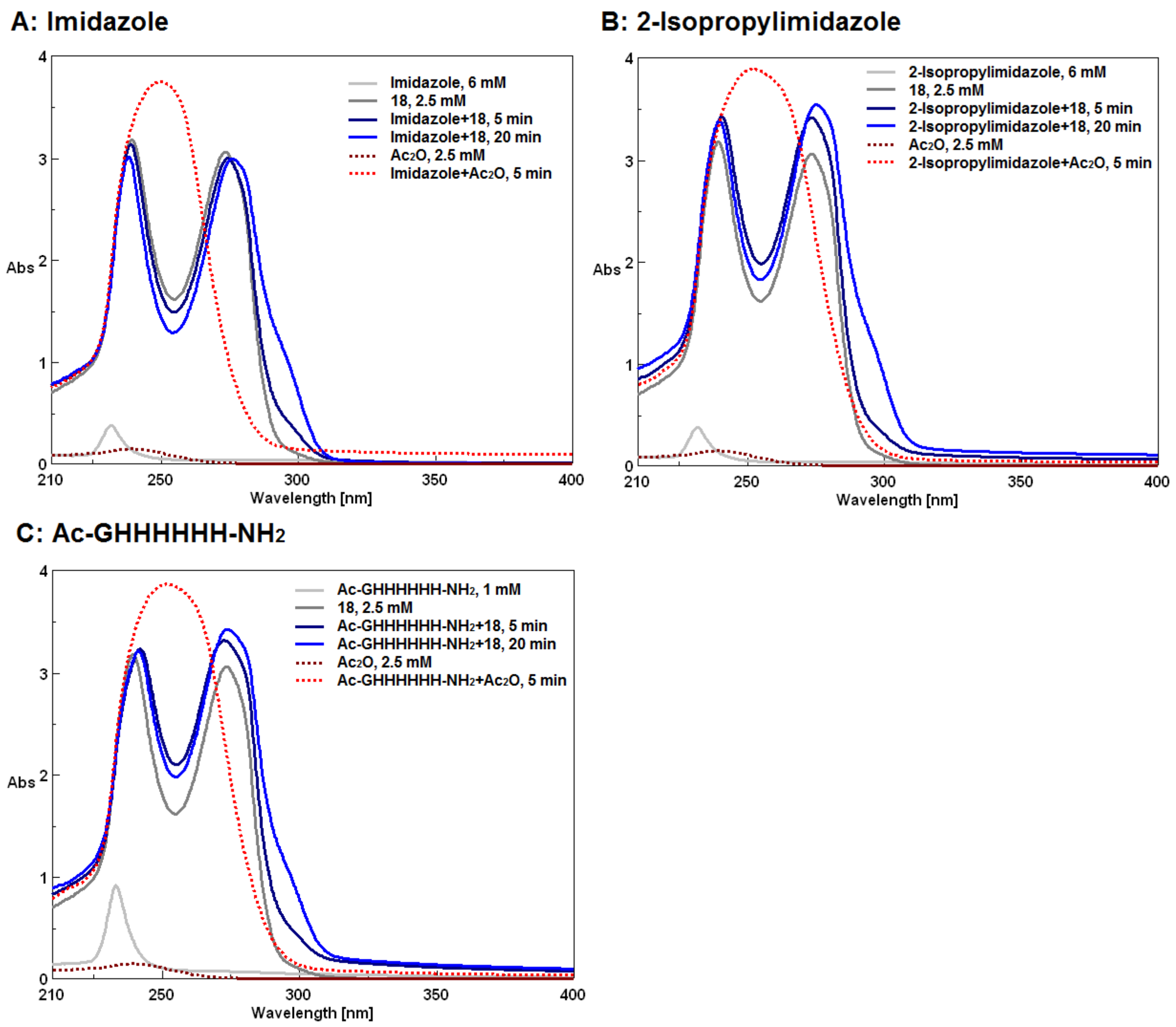

Supplementary Figure 21. Spectrophotometric analysis of potential N-acyl imidazole formation. Reactions with A) imidazole, B) 2-isopropylimidazole, and C) Ac-GHHHHHH-NH_2 at 25 °C. The characteristic absorption of N-acyl imidazoles at 245 nm was evident with acetic anhydride (red dashed lines), but it was not detected when **18** was used as the acylating agent (blue lines). A solution of the imidazole (6 mM for imidazole and 2-isopropylimidazole, or 1 mM for Ac-GHHHHHH-NH_2) was prepared in 200 mM HEPES buffer, pH 7.5. To 0.450 mL of this solution was added 0.05 mL of a 25 mM solution of the acylating agent (**18** or acetic anhydride) to provide a final concentration of 2.5 mM. Following rapid mixing, UV spectra were acquired using a Jasco V-650 spectrophotometer.

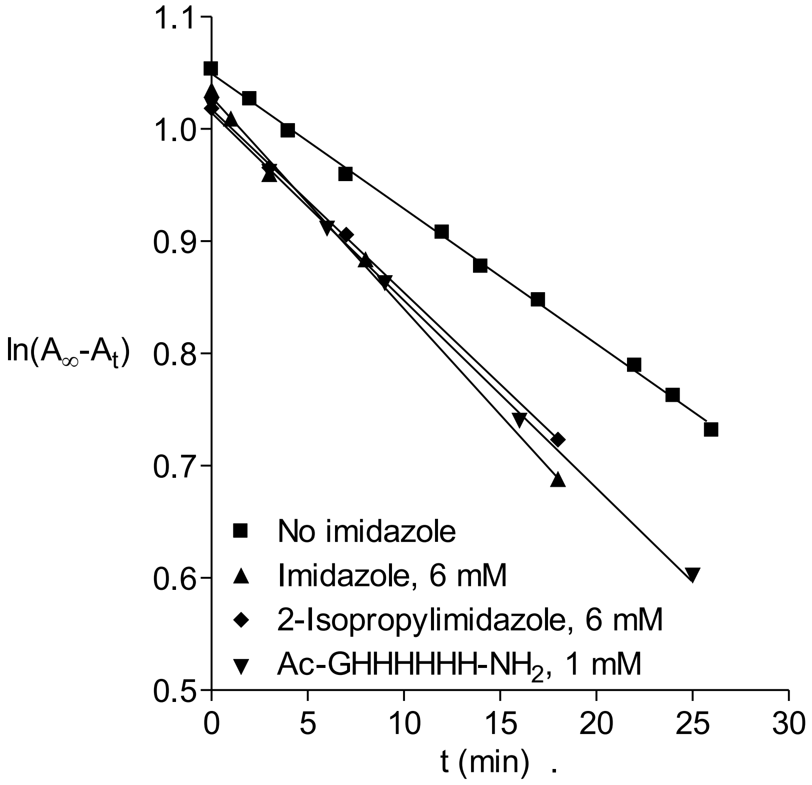

Supplementary Figure 22. Hydrolysis of **18** in the presence of different imidazoles. The observed hydrolysis rate of **18** with 6 mM 2-isopropylimidazole was essentially identical to that of 6 mM imidazole or 1 mM Ac-GHHHHHH-NH_2 in HEPES buffer, pH 7.5, at 25 °C. The absorption at 295 nm of 4-methoxyphenol was used in the kinetic determinations of hydrolysis rates. Conditions were identical to those reported above in Supplementary Fig. 21. Curves are based on single measurements.

Proton NMR (300 MHz, chloroform-*d*, compound **16**)

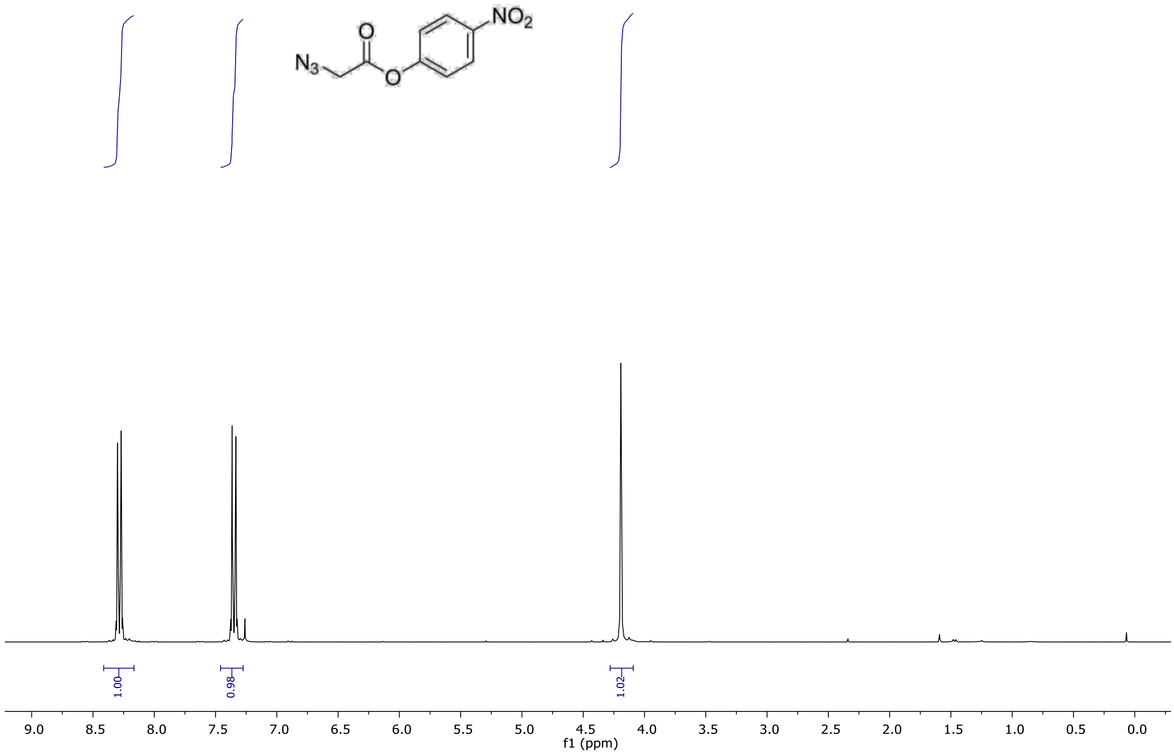

Carbon NMR (75 MHz, chloroform-*d*, compound **16**)

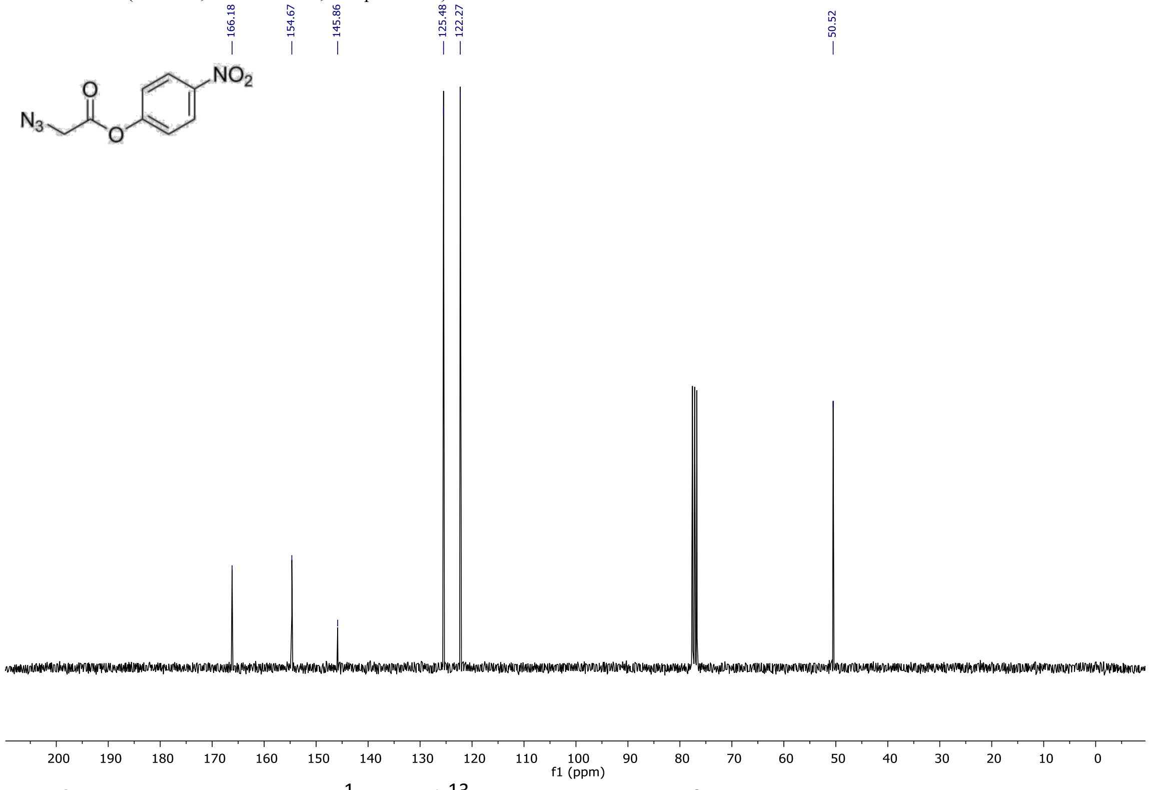

Supplementary Figure 23. ^{1}H- and ^{13}C-NMR spectra of ester **16**.

Proton NMR (300 MHz, chloroform-*d*, compound **17**)

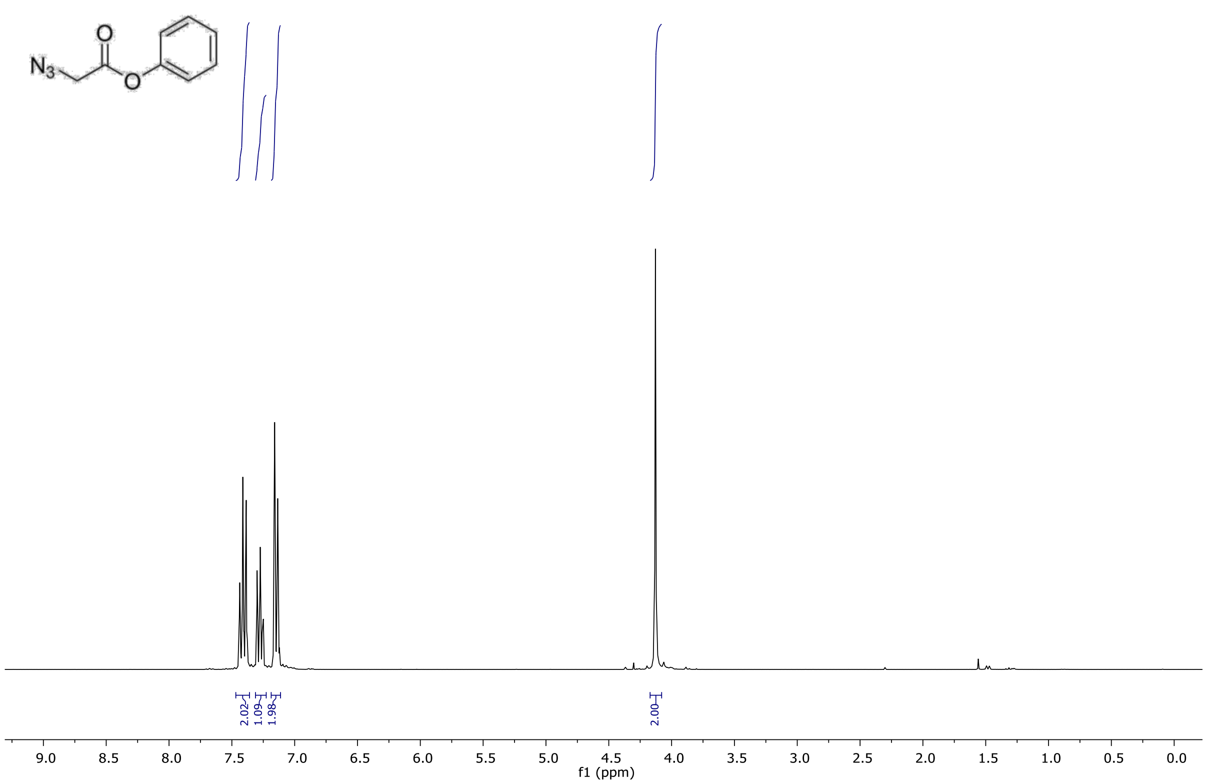

Carbon NMR (75 MHz, chloroform-*d*, compound **17**)

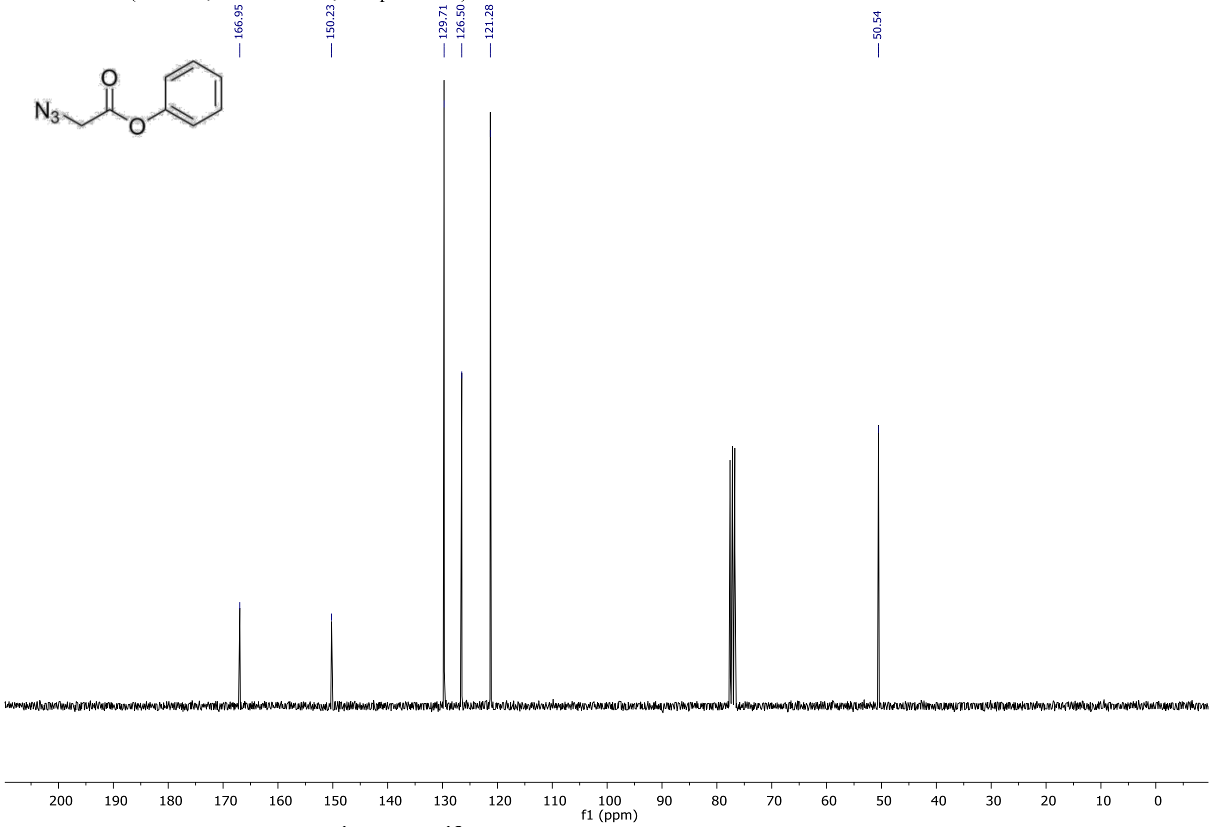

Supplementary Figure 24. ^{1}H- and ^{13}C-NMR spectra of ester **17**.

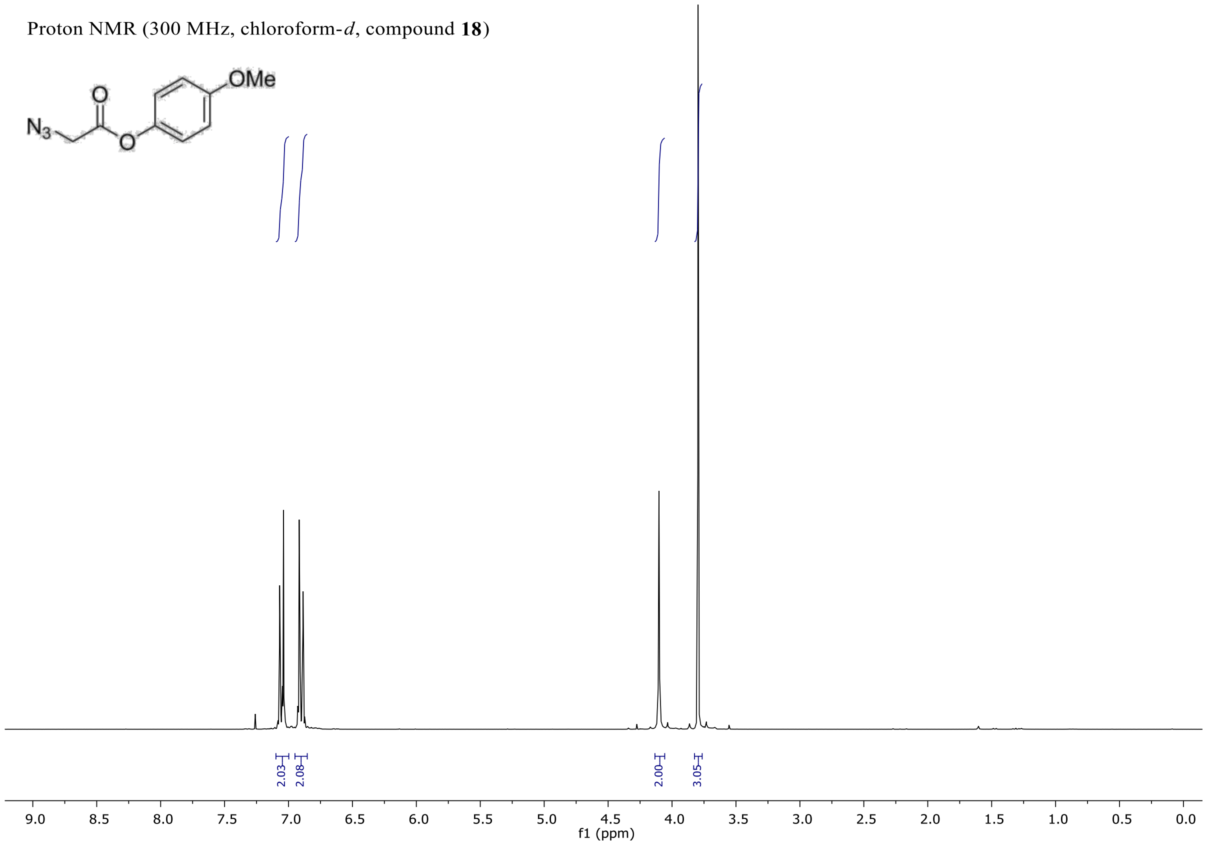

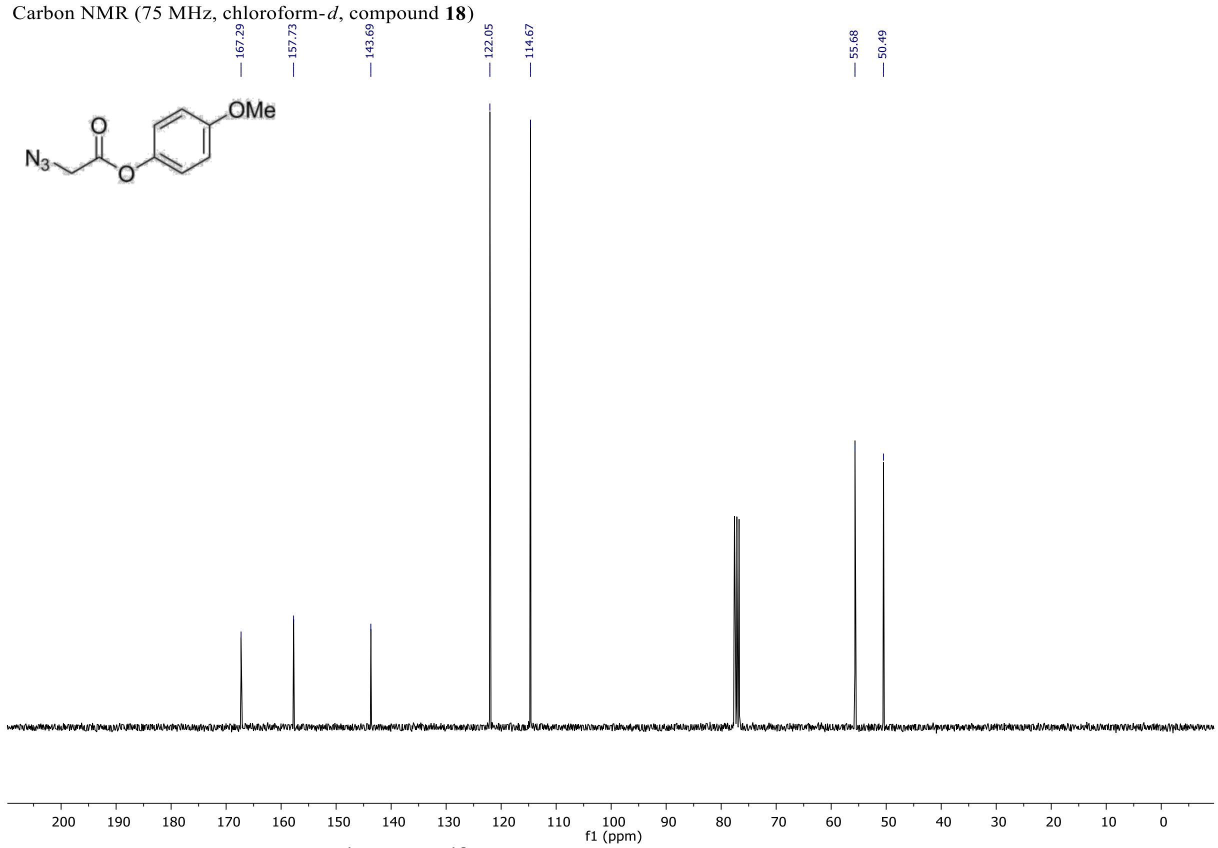

Supplementary Figure 25. ^{1}H- and ^{13}C-NMR spectra of ester **18**.

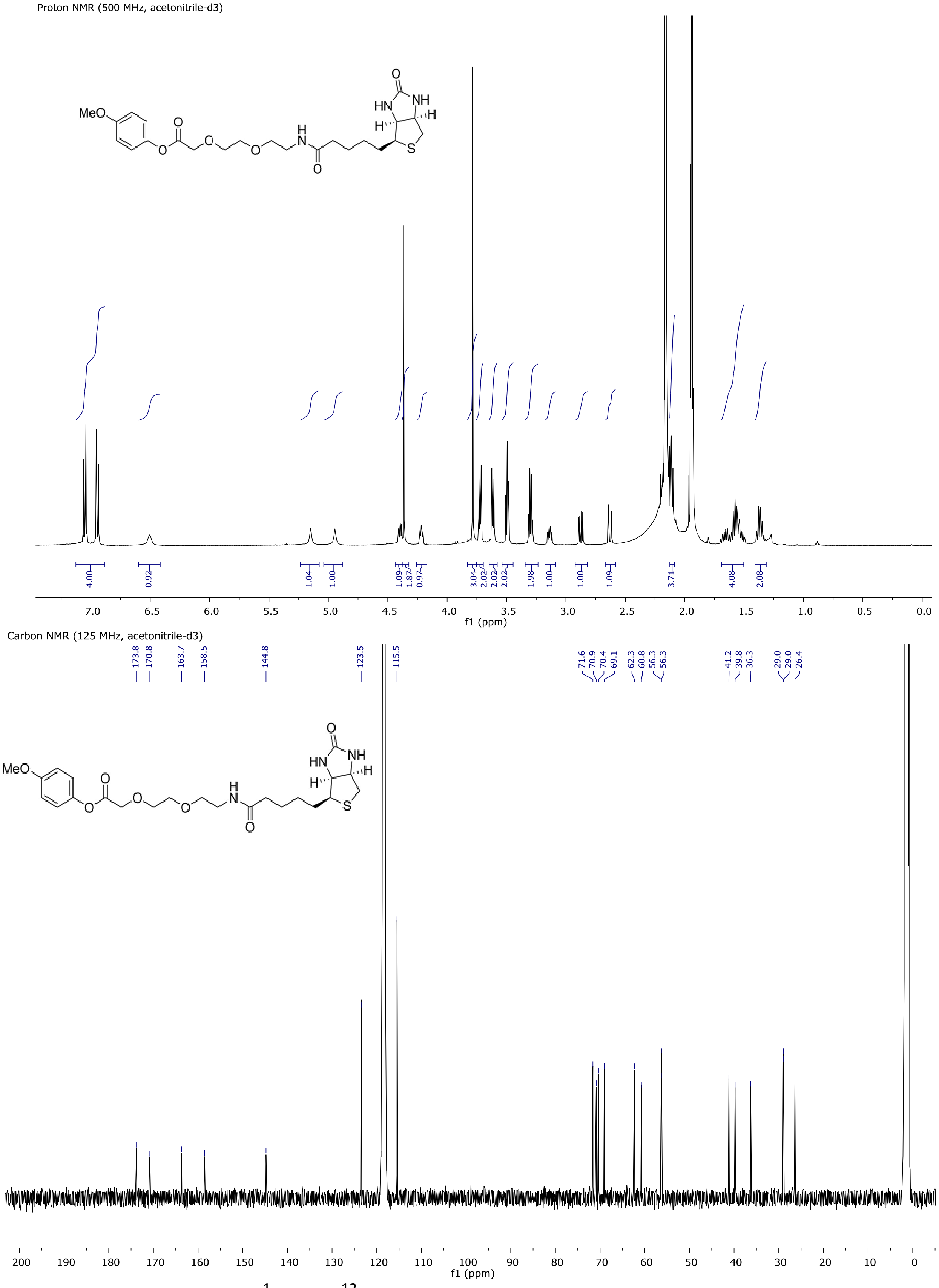

Supplementary Figure 26. ^{1}H- and ^{13}C-NMR spectra of biotin reagent **20**.

COSY spectrum (acetonitrile-d3)

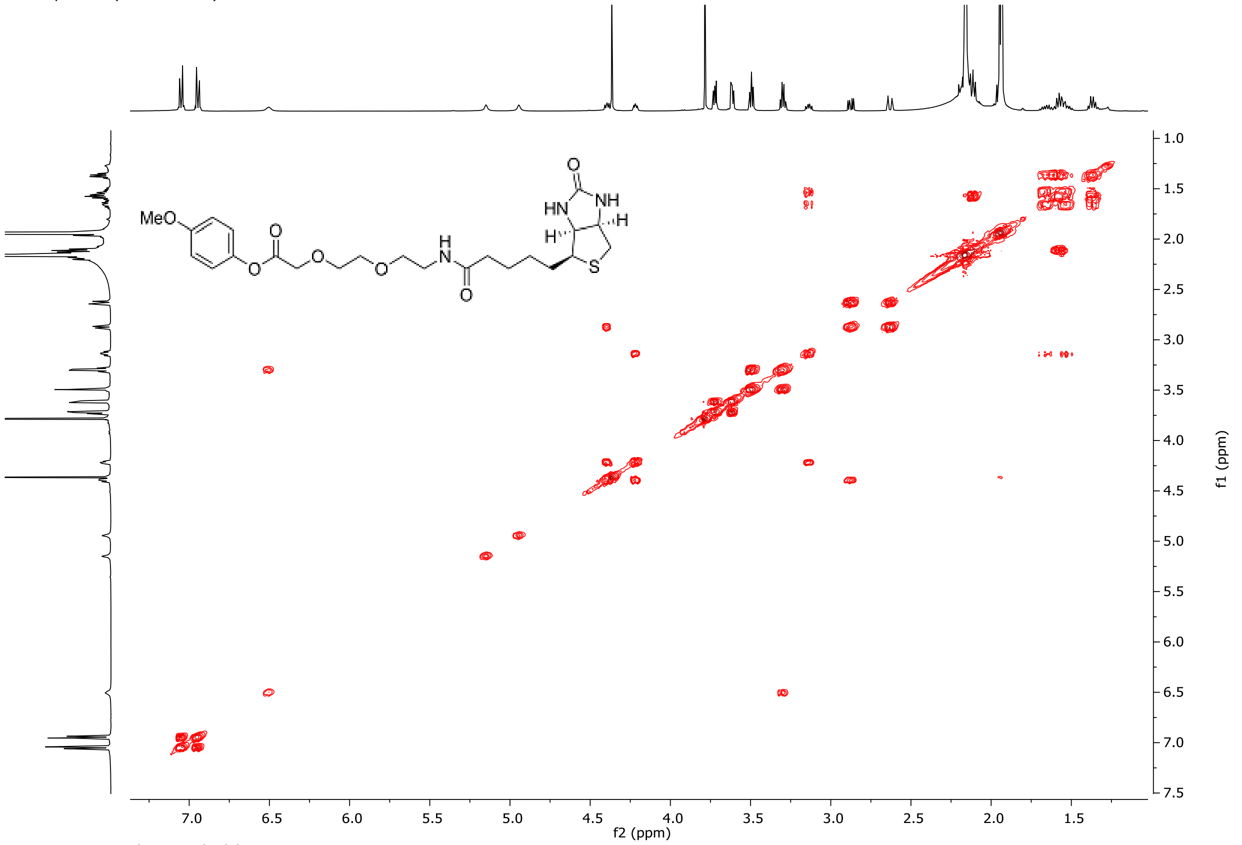

HSQC spectrum (acetonitrile-d3)

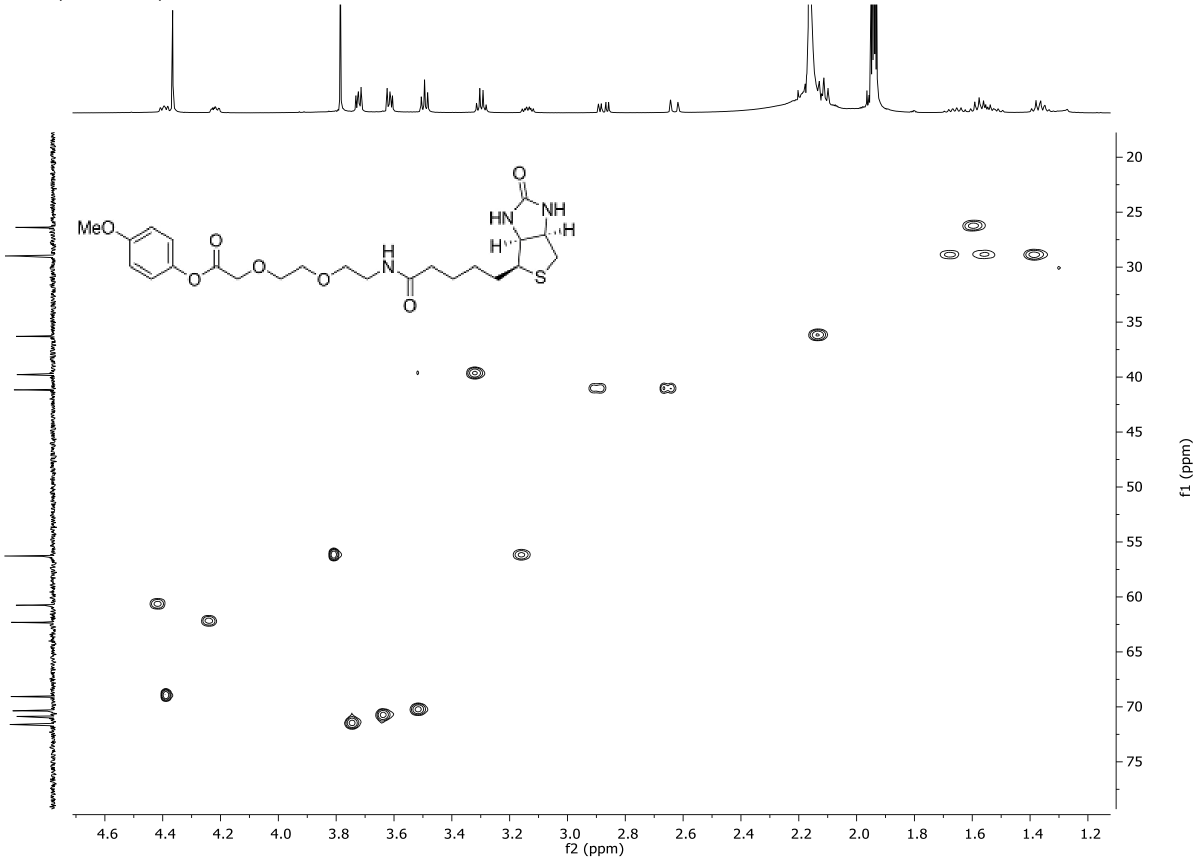

Supplementary Figure 27. COSY and HSQC NMR spectra of biotin reagent **20**.

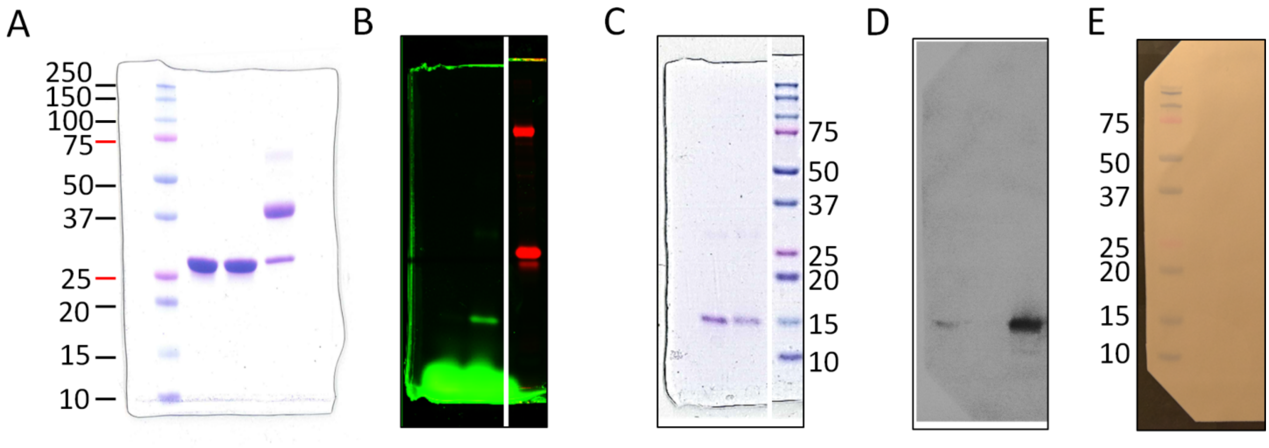

Supplementary Figure 28. Uncropped versions of the gels and blots depicted in Figures 4 and 5. The Dual Color Precision Plus Protein™ Standard (BioRad) was used in all cases. A) Coomassie-stained SDS-PAGE gel of the two-step PEGylation of GH_6-EGFP. B) Fluorescence image of the SDS-PAGE gel of the fluorescent labeling of GH_6-BIR2. Lane 3–10 and lane 12–15 are not shown, as they contained samples unrelated to the research described in the present paper. The fluorescent bands in lane 11 (the protein standard) correspond to the red-colored 25- and 75-kDa size markers. C) Image of the same gel as depicted in B) after Coomassie staining. D) Western blot of the biotinylation of GH_6-BIR2. E) Photographic image of the same blot, showing the location of the size markers in the left-most lane.

Supplementary Tables

Supplementary Table 1. Optimization of the acylation of GH_6-SUMO with ester **18**[a]

Entry	Equiv.	Temp. (°C)	Time (h)	% mono	% di	% unreacted
1	2.5	4	1	6	-	94
2	2.5	4	16	39	3	58
3	2.5+2.5	4	16+4	41	3	56
4	2.5+2.5	4	16+16	60	4	36
5	10	4	16	70	5	25
6	10	25	1.5	25	1	75
7	10	25	3	38	2	60
8	20	4	16	74	6	20
9	20	25	1.5	37	2	60
10	20	25	3	53	3	44
11	20	25	9	76	7	17
12	10+10	25	3+0.5	59	5	35
13	10+10	25	3+6	80	6	14
14	40	4	1	46	3	50
15	40	4	36	65	25	10

[a] Conversion of GH_6-SUMO (154 µM) after treatment with varying amounts of ester **18** in 200 mM HEPES buffer at pH 7.5 at 4 °C or room temperature, based on the relative abundance of the different species in the deconvoluted ESI-MS spectra.

Supplementary Table 2. Purity and masses of all peptides synthesized.

Compound	Sequence[a]	Chemical Formula	Purity[a]	Calculated Mass	Found
**2**	H-LRFKFY-NH$_2$	$C_{45}H_{65}N_{11}O_7$	95 %	871.5	$[M+H]^+$ 872.5
**3**	H-GLRFKFY-NH$_2$	$C_{47}H_{68}N_{12}O_8$	97%	928.5	$[M+2H]^{2+}$ 465.3
**4**	H-GSLRFKFY-NH$_2$	$C_{50}H_{73}N_{13}O_{10}$	96%	1015.6	$[M+2H]^{2+}$ 508.8
**5**	H-HLRFKFY-NH$_2$	$C_{51}H_{71}N_{13}O_9$	95 %	1009.5	$[M+2H]^{2+}$ 505.3
**6**	H-HHHLRFKFY-NH$_2$	$C_{63}H_{86}N_{20}O_{10}$	97 %	1282.6	$[M+3H]^{3+}$ 428.9
**7**	H-GHLRFKFY-NH$_2$	$C_{53}H_{75}N_{15}O_9$	96%	1065.6	$[M+3H]^{3+}$ 356.2
**8**	H-GSHLRFKFY-NH$_2$	$C_{56}H_{80}N_{16}O_{11}$	94 %	1152.6	$[M+H]^+$ 1153.6
**9**	H-GHHLRFKFY-NH$_2$	$C_{59}H_{82}N_{18}O_{10}$	96%	1202.6	$[M+2H]^{2+}$ 602.3
**10**	H-GHHHLRFKFY-NH$_2$	$C_{65}H_{89}N_{21}O_{11}$	84 %	1339.7	$[M+H]^+$ 1340.7
**11**	H-AHHHLRFKFY-NH$_2$	$C_{66}H_{91}N_{21}O_{11}$	96 %	1353.7	$[M+H]^+$ 1355.8
**12**	H-VHHHLRFKFY-NH$_2$	$C_{68}H_{93}N_{21}O_{11}$	96 %	1381.8	$[M+H]^+$ 1382.9
**13**	H-Beltide-1	$C_{106}H_{158}N_{24}O_{27}$	99 %	2199.1728	$[M+2H]^{2+}$ 1100.5670; $[M+3H]^{3+}$ 734.0477; $[M+4H]^{4+}$ 550.7849
**13Ac**	Ac-Beltide-1	$C_{108}H_{160}N_{24}O_{28}$	99 %	2241.1834	$[M+2H]^{2+}$ 1121.6032; $[M+3H]^{3+}$ 748.0725; $[M+4H]^{4+}$ 561.3043
**14**	H-GHHH-Beltide-1	$C_{126}H_{182}N_{24}O_{28}$	98 %	2667.3710	$[M+2H]^{2+}$ 1334.6865; $[M+3H]^{3+}$ 890.1273; $[M+4H]^{4+}$ 667.8472
**15**	H-GHHHHHH-Beltide-1	$C_{144}H_{203}N_{43}O_{34}$	99 %	3078.5478	$[M+3H]^{3+}$ 1027.1925; $[M+4H]^{4+}$ 769.6462; $[M+5H]^{5+}$ 616.7181
**15G**	H-GHHHHHHG-Beltide-1	$C_{146}H_{206}N_{44}O_{35}$	99 %	3135.5692	$[M+3H]^{3+}$ 1046.1855; $[M+4H]^{4+}$ 784.8909; $[M+5H]^{5+}$ 628.1135
**15K**	H-GHHHHHHK-Beltide-1	$C_{150}H_{215}N_{45}O_{35}$	99 %	3206.6427	$[M+3H]^{3+}$ 1070.2005; $[M+4H]^{4+}$ 802.9025; $[M+5H]^{5+}$ 642.5233
**15Ac**	Ac-GHHHHHH-Beltide-1	$C_{146}H_{205}N_{43}O_{35}$	99 %	3120.5583	$[M+3H]^{3+}$ 1041.2023; $[M+4H]^{4+}$ 781.1532; $[M+5H]^{5+}$ 625.1244
**15K-Ac**	Ac-GHHHHHHK-Beltide-1	$C_{152}H_{217}N_{45}O_{36}$	99 %	3248.6533	$[M+3H]^{3+}$ 1084.2048; $[M+4H]^{4+}$ 813.4057; $[M+5H]^{5+}$ 650.9259
-	Ac-GHHHHHH-NH$_2$	$C_{40}H_{50}N_{20}O_8$	99%	938.4	$[M+H]^+$ 939.4144; $[M+2H]^{2+}$ 470.2156; $[M+3H]^{3+}$ 313.8122

[a] Beltide-1 amino acid sequence: DWLKAFYDKVAEKLKEAF

[b] Purity based on HPLC UV trace at 215 nm

Supplementary Table 3. Mass spectrometry data of acylated Beltide-1 derivatives

Compound	Sequence[a]	Chemical Formula	Calculated Mass	Found
**14Gdl**	D-Gluconoyl-GHHH-Beltide-1	$C_{132}H_{192}N_{34}O_{37}$	2845.4188	$[M+2H]^{2+}$ 1424.2; $[M+3H]^{3+}$ 949.9; $[M+4H]^{5+}$ 712.6
**15Gdl**	D-Gluconoyl-GHHHHHH-Beltide-1	$C_{150}H_{213}N_{43}O_{40}$	3256.5955	$[M+2H+Na]^{3+}$ 1094.8681; $[M+3H+Na]^{4+}$ 821.4030; $[M+4H+Na]^{5+}$ 657.3236
**14Az**	2-Azidoacetyl-GHHH-Beltide-1	$C_{128}H_{183}N_{37}O_{32}$	2750.3830	$[M+2H]^{2+}$ 1376.7; $[M+3H]^{3+}$ 918.0; $[M+4H]^{4+}$ 688.7
**15Az**	2-Azidoacetyl-GHHHHHH-Beltide-1	$C_{146}H_{204}N_{46}O_{35}$	3161.5597	$[M+3H]^{3+}$ 1054.8588; $[M+4H]^{4+}$ 791.3983; $[M+4H+Na]^{5+}$ 633.3192
**15Az-Az**	2-Azidoacetyl-GHHHHHH-Beltide-1(2-azidoacetyl)	$C_{148}H_{205}N_{49}O_{36}$	3244.5717	$[M+3H]^{3+}$ 1082.8475; $[M+4H]^{4+}$ 812.3879; $[M+5H]^{5+}$ 650.1115
**15G-Az**	2-Azidoacetyl-GHHHHHHG-Beltide-1	$C_{150}H_{213}N_{43}O_{40}$	3218.5812	$[M+3H]^{3+}$ 1073.8681; $[M+4H]^{4+}$ 805.6527; $[M+5H]^{5+}$ 644.7236
**15K-Az**	2-Azidoacetyl-GHHHHHHK-Beltide-1	$C_{152}H_{216}N_{48}O_{36}$	3289.6547	$[M+3H]^{3+}$ 1097.8782; $[M+4H]^{4+}$ 823.6610; $[M+5H]^{5+}$ 659.1302
**15K-Az-Az**	2-Azidoacetyl-GHHHHHHK-Beltide-1(2-azidoacetyl)	$C_{154}H_{217}N_{51}O_{37}$	3372.6666	$[M+3H]^{3+}$ 1125.5474; $[M+4H]^{4+}$ 844.4131; $[M+5H]^{5+}$ 675.7322
**15Ac-Az**	Ac-GHHHHHH-Beltide-1(2-azidoacetyl)	$C_{148}H_{206}N_{46}O_{36}$	3203.5703	$[M+3H]^{3+}$ 1069.1872; $[M+4H]^{4+}$ 801.8918; $[M+6H]^{6+}$ 535.5992
**15K-Ac-Az**	Ac-GHHHHHHK(2-azidoacetyl)-Beltide-1	$C_{154}H_{218}N_{48}O_{37}$	3331.6653	$[M+3H]^{3+}$ 1111.8791; $[M+4H]^{4+}$ 834.1617; $[M+5H]^{5+}$ 667.5310

[a] Beltide-1 amino acid sequence: DWLKAFYDKVAEKLKEAF

Supplementary Methods

Protein sequences

Lys residues are underscored.

GH$_6$-EGFP (Calculated M_r 30774.39 Da)
GHHHHHHSSGLVPRGSHMLEKREAEAGRLGAGGPVATMVSKGEELFTGVVPILVELDGDVNGHKFSVSGEGE
GDATYGKLTLKFICTTGKLPVPWPTLVTTLTYGVQCFSRYPDHMKQHDFFKSAMPEGYVQERTIFFKDDGNYKT
RAEVKFEGDTLVNRIELKGIDFKEDGNILGHKLEYNYNSHNVYIMADKQKNGIKVNFKIRHNIEDGSVQLADHYQ
QNTPIGDGPVLLPDNHYLSTQSALSKDPNEKRDHMVLLEFVTAAGITLGMDELYK

GSSH$_6$-EGFP (Calculated M_r 30948.55 Da)
GSSHHHHHHSSGLVPRGSHMLEKREAEAGRLGAGGPVATMVSKGEELFTGVVPILVELDGDVNGHKFSVSGEG
EGDATYGKLTLKFICTTGKLPVPWPTLVTTLTYGVQCFSRYPDHMKQHDFFKSAMPEGYVQERTIFFKDDGNYK
TRAEVKFEGDTLVNRIELKGIDFKEDGNILGHKLEYNYNSHNVYIMADKQKNGIKVNFKIRHNIEDGSVQLADHY
QQNTPIGDGPVLLPDNHYLSTQSALSKDPNEKRDHMVLLEFVTAAGITLGMDELYK

GSH-EGFP (Calculated M_r 29197.71 Da)
GSHMLEKREAEAGRLGAGGPVATMVSKGEELFTGVVPILVELDGDVNGHKFSVSGEGEGDATYGKLTLKFICTT
GKLPVPWPTLVTTLTYGVQCFSRYPDHMKQHDFFKSAMPEGYVQERTIFFKDDGNYKTRAEVKFEGDTLVNRIE
LKGIDFKEDGNILGHKLEYNYNSHNVYIMADKQKNGIKVNFKIRHNIEDGSVQLADHYQQNTPIGDGPVLLPDN
HYLSTQSALSKDPNEKRDHMVLLEFVTAAGITLGMDELYK

GH$_6$-MBP (Calculated M_r 41088.48 Da)
GHHHHHHKIEEGKLVIWINGDKGYNGLAEVGKKFEKDTGIKVTVEHPDKLEEKFPQVAATGDGPDIIFWAHDRF
GGYAQSGLLAEITPDKAFQDKLYPFTWDAVRYNGKLIAYPIAVEALSLIYNKDLLPNPPKTWEEIPALDKELKAKGK
SALMFNLQEPYFTWPLIAADGGYAFKYENGKYDIKDVGVDNAGAKAGLTFLVDLIKNKHMNADTDYSIAEAAF
NKGETAMTINGPWAWSNIDTSKVNYGVTVLPTFKGQPSKPFVGVLSAGINAASPNKELAKEFLENYLLTDEGLE
AVNKDKPLGAVALKSYEEELAKDPRIAATMENAQKGEIMPNIPQMSAFWYAVRTAVINAASGRQTVDEALKDA
QT

GH$_6$-SUMO (Calculated M_r 12476.90 Da)
GHHHHHHMSDSEVNQEAKPEVKPEVKPETHINLKVSDGSSEIFFKIKKTTPLRRLMEAFAKRQGKEMDSLRFLY
DGIRIQADQTPEDLDMEDNDIIEAHREQIGGATY

GH$_6$-BIR2 (Calculated M_r 15550.12 Da)
GHHHHHHSSGLVPRGSHMRDHFALDRPSETHADYLLRTGQVVDISDTIYPRNPAMYSEEARLKSFQNWPDYA
HLTPRELASAGLYYTGIGDQVQCFACGGKLKNWEPGDRAWSEHRRHFPNCFFVLGRNLNIRSE

Reaction of GH_6-EGFP with biotin derivative 21

1 µL of a 25 mM solution of 4-methoxyphenyl 2-azidoacetate **18** in acetonitrile was mixed with 5 µL of a 5 mM solution of DBCO-PEG4-Biotin **19** in CH_3CN/H_2O 1:1 and the resulting solution was incubated at room temperature for 15 min. After that time, LC-MS showed full conversion to compound **21**. 5 µL of this solution (4.2 mM **21** in CH_3CN/H_2O 7:5) were added to 10 µL of a 35 µM solution of GH_6-EGFP in 200 mM HEPES buffer at pH 7.5 and the resulting solution was incubated at 4 ^{o}C. The progress of the reaction was followed by LC-MS.
